# Pan-cancer analysis predict that FAT1 is a therapeutic target and immunotherapy biomarker for multiple cancer types including non-small cell lung cancer

**DOI:** 10.3389/fimmu.2024.1369073

**Published:** 2024-05-24

**Authors:** Chen Ding, Hua Huang, Di Wu, Chen Chen, Yu Hua, Jinghao Liu, Yongwen Li, Hongyu Liu, Jun Chen

**Affiliations:** ^1^ Department of Lung Cancer Surgery, Tianjin Medical University General Hospital, Tianjin, China; ^2^ Tianjin Key Laboratory of Lung Cancer Metastasis and Tumor Microenvironment, Tianjin Lung Cancer Institute, Tianjin Medical University General Hospital, Tianjin, China

**Keywords:** FAT1, pan-cancer, prognosis, bioinformatics, immune

## Abstract

FAT1, a substantial transmembrane protein, plays a pivotal role in cellular adhesion and cell signaling. Numerous studies have documented frequent alterations in FAT1 across various cancer types, with its aberrant expression being linked to unfavorable survival rates and tumor progression. In the present investigation, we employed bioinformatic analyses, as well as *in vitro* and *in vivo* experiments to elucidate the functional significance of FAT1 in pan-cancer, with a primary focus on lung cancer. Our findings unveiled FAT1 overexpression in diverse cancer types, including lung cancer, concomitant with its association with an unfavorable prognosis. Furthermore, FAT1 is intricately involved in immune-related pathways and demonstrates a strong correlation with the expression of immune checkpoint genes. The suppression of FAT1 in lung cancer cells results in reduced cell proliferation, migration, and invasion. These collective findings suggest that FAT1 has potential utility both as a biomarker and as a therapeutic target for lung cancer.

## Introduction

Cancer stands as a prominent global cause of mortality. Among various cancer types, lung cancer emerges as the primary contributor to cancer-related fatalities (1). Non-small cell lung cancer (NSCLC) constitutes approximately 85% of all lung cancer cases, making it the most prevalent histological subtype. Despite significant advancements in our understanding of the biological foundations of NSCLC, the integration of predictive biomarkers, and the refinement of treatment strategies, substantial progress has been achieved over the past two decades, resulting in improved outcomes for numerous patients (2). However, individuals afflicted with advanced-stage disease still confront a grim prognosis. Hence, the identification of novel biomarkers and therapeutic targets capable of enhancing patient outcomes represents an urgent imperative.

FAT atypical cadherin 1 (FAT1), a gene encoding protocadherin, ranks among the most frequently mutated genes in human cancer. As a transmembrane protein, FAT1 assumes a pivotal role in cellular adhesion and signaling pathways (3). Located on chromosome 4q35, it functions as a tumor-promoting gene, exerting regulatory control over cell proliferation, migration, and invasion (4–7). Notably, FAT1 undergoes frequent alterations in various cancer types, including lung cancer, where its mutations correlate with unfavorable survival rates and tumor progression (8, 9). In a prior study, our research unveiled the potential of FAT1 mutations as predictive biomarkers in NSCLC, aiding in the identification of patients less likely to derive sustained clinical benefits from immune checkpoint blockade (ICB). We proposed an FAT1 mutation-based model for the screening of NSCLC patients more suitable for ICB, thereby contributing to individualized immunotherapy (10). Recent investigations have further illuminated the role of FAT1 alterations in multiple signaling pathways and critical cellular processes, such as the Hippo and Wnt signaling pathways, along with epithelial–mesenchymal transition (EMT), all of which play pivotal roles in tumorigenesis (11). Studies exploring FAT1’s significance in cancer have examined its prognostic value, association with immune infiltration, and impact on the tumor microenvironment (12, 13). However, the functional implications of FAT1 alterations across various cancer types, as well as their potential as therapeutic biomarkers, require further elucidation.

This study employs bioinformatic methodologies, as well as the *in vitro* and *in vivo* experiments to explore the functional role of FAT1 across NSCLC, aiming to elucidate its expression patterns, genetic alterations, and functional networks. Our analysis provides a comprehensive examination of FAT1, aiming to elucidate the potential mechanisms through which FAT1 mediates tumorigenesis and assess its clinical relevance in a pan-cancer context. This encompasses an in-depth exploration of FAT1’s biological role in non-small cell lung cancer, with a focus on investigating its impact on cell proliferation and migration, revealing its potential significance in the progression of NSCLC.

## Materials and methods

### NSCLC samples and next-generation sequencing

We collected 37 fresh tumor tissues from patients who were diagnosed with NSCLC at Tianjin Medical University General Hospital. Tissues were collected by surgery, and routinely processed with formalin fixation, embedded with paraffin. All cases were confirmed postoperative histological pathology with NSCLC. All patients provided written consent, and the research was approved by the Institutional Ethics Committee of the General Hospital of Tianjin Medical University. Tumor DNA was extracted from 5 to 10 10μm FFPE curls, and DNA quantification was performed using Qubit™ dsDNA HS and BR Assay Kits (Thermo Fisher Scientific, MA, USA). Target gene capture NGS technology was employed to detect 1267 genes related to lung cancer treatment plans, high-throughput sequencing data were obtained, and somatic mutations were identified by comparing with matched adjacent lung tissues. The NGS sequencing process was completed by Yuce Biological Technology Co., Ltd.

### Data sources and processing for FAT1 mRNA expression

FAT1 expression was analyzed in 34 different tumor types and their corresponding normal tissues using a combination of The Cancer Genome Atlas (TCGA) and GTEx cohorts. For this analysis, SangerBox, a web-based tool (http://sangerbox.com/), was utilized to obtain FAT1 expression levels in different pathological stages (stages I–IV) of selected TCGA tumors through the “Pathological Stage Plot” module of Gene Expression Profiling Interactive Analysis. Violin plots were generated to depict the relationship between FAT1 expression levels and pathological stages. Survival analysis was performed using the Kaplan–Meier method and Cox proportional hazards regression analysis. Furthermore, protein expression levels were investigated using the UALCAN portal (http://ualcan.path.uab.edu/index.html) (14), which offers an interactive web resource for CPTAC analysis. The UALCAN portal utilizes the CPTAC database and normalizes logged expression values to standard deviations from the median in each proteomic profile.

### Genetic analysis

To explore FAT1 genetic alterations in various cancer types, we analyzed somatic mutation data retrieved from cBioPortal (available at https://www.cbioportal.org/) (15). Through this analysis, information regarding the frequency and types of mutations in FAT1 across cancer types was acquired.

### FAT1-related gene enrichment analysis

To conduct gene enrichment analysis related to FAT1, the STRING website was utilized (available at https://string-db.org/) (16). Furthermore, the “Similar Gene Detection” module of GEPIA2 was employed to generate a list of the top 100 FAT1-associated genes in TCGA tumors. Subsequently, the correlation analysis module of GEPIA2 was employed to explore the correlation between FAT1 and these top five FAT1-associated targeting genes. To perform pathway and process enrichment analysis for the identified top 100 FAT1-associated targeting genes, the Metascape web-based tool (17) was utilized, and specific parameters were selected, including P < 0.01, a minimum count of three for the terms, and an enrichment factor > 1.5 for canonical pathways.

### Analysis of tumor immune and immunosuppressive cell infiltration

By using the TIMER2 server, we examined the correlation between FAT1 expression and the infiltration of various immune cell types. To evaluate the effect of genetic and epigenetic alterations of FAT1 on dysfunctional T-cell phenotypes, the QUERY module of the Tumor Immune Dysfunction and Exclusion (TIDE) algorithm was utilized (18).

### Epigenetic methylation analysis

To examine the differences in FAT1 methylation levels between tumor and paired normal tissues across various TCGA cancer types, the TCGA methylation module within the UALCAN interactive web resource was employed (19, 20). Furthermore, the TIDE server was utilized to investigate the effect of FAT1 methylation on dysfunctional T-cell phenotypes and prognoses.

### Analysis of gene expression correlations with therapeutic responses

To assess the therapeutic potential of FAT1 as a target in various cancers, the drug sensitivity data obtained from the ROC Plotter was examined (http://www.rocplot.org/). The ROC Plotter is a transcriptome-based tool that enables the prediction of biomarkers by establishing connections between gene expressions and responses to therapy among patients with cancer (21).

### Cell culture and transfection

The human cancer cell lines OVCAR3, Hep3B, PANC1, H1299, A549, as well as the embryonic kidney 293T were obtained from the American Type Culture Collection. These cell lines were cultured in DMEM supplemented with 10% fetal bovine serum and 1% penicillin–streptomycin in a humidified incubator at 37°C with 5% CO_2_. To perform the transfection, siRNA specifically targeting FAT1 or a negative control siRNA (Ribobio, China) was introduced into the cells using Lipofectamine 2000 (Invitrogen, USA) following the manufacturer’s instructions. The siRNAs targeting FAT1 were sense: 5′-GCACCACAAUUUCGAGCAATT-3′, antisense: 5′-UUGCUCGAAAUUGUGGUGCTT-3′.

### Real-time polymerase chain reaction

According to the manufacturer’s instructions, the cells were digested down with trypsin, and the total RNA was extracted using the SPARKeasy Cell RNA Rapid Extraction Kit (Sparkjade, Shandong, China). RNA concentration was measured, and a total of 2 μg of RNA was reverse-transcribed into complementary DNA using a reverse-transcription kit (Takara, Beijing, China) according to the manufacturer’s instructions. Real-time PCR used a fully automated PCR analyzer SLAN-96P, with GAPDH as the internal reference. The mRNA primer sequences used were as follows: 5′-GGAGCGAGATCCCTCCAAAAT-3′ and 5′- GGCTGTTGTCATACTTCTCATGG -3′ for GAPDH; 5′-CATCCTGTCAAGATGGGTGTTT-3′ and 5′- TCCGAGAATGTACTCTTCAGCTT-3′ for FAT1.

### Cell proliferation assay

Cell proliferation was assessed using the Cell Counting Kit-8 (CCK8) assay. Briefly, cells were seeded into 96-well plates and incubated for 24, 48, and 72 h. At each time point, 10 μL of the CCK8 solution was added to each well and incubated for 2 h. The absorbance was measured at 450 nm using a microplate reader.

### 5-ethynyl-2’-deoxyuridine assay

Cell proliferation was also assessed using the EdU assay. Briefly, the cells were subsequently stained with cell-Light™ EdU Apollo567 *In Vitro* Kit (Ribobio, Guangzhou, China) following the manufacturer’s instructions. The images were captured using a fluorescence microscope, and the percentage of EdU-positive cells was calculated using ImageJ.

### Colony formation assay

The cells were seeded into 6-well plates at a density of 500 cells per well, cultured for 14 days, and fixed with 4% paraformaldehyde and stained with crystal violet. The area of colonies was counted using ImageJ.

### Transwell assay

Cell migration and invasion were evaluated using Transwell chambers. For the migration assay, cells were seeded into the upper chamber with a serum-free medium, whereas the lower chamber was filled with a medium containing 10% fetal bovine serum. After incubation for 24 h, the cells that migrated to the lower chamber were fixed with 4% paraformaldehyde and stained with crystal violet. For the invasion assay, Transwell chambers were coated with 20% Matrigel before cell seeding.

### Scratch wound-healing assay

The cells were seeded into 6-well plates and cultured to confluence. A scratch wound was created using a sterile 200 μL pipette tip, and the cells were washed with phosphate-buffered saline to remove the debris. Then, the cells were incubated in serum-free medium, and wound closure was monitored at different time points using an inverted microscope.

### Protein extraction and western blot analysis

Total protein was extracted, and protein concentration was measured by the BCA method. The proteins to be isolated were separated with 8% to 10% sodium dodecyl sulfate-polyacrylamide gel electrophoresis, transferred to polyvinylidene fluoride membrane, and incubated overnight at 4°C under the following primary antibodies: anti-β-TUBULIN (66240–1-Ig, Proteintech), anti-FAT1 (E95869, Sigma), anti-YAP (13584–1-AP, Proteintech), anti-P-TAZ (AF4315, Affinity), Anti-FAK (12636–1-AP, Proteintech), and anti-Src (11097–1-AP, Proteintech). Then, the membrane was incubated with anti-rabbit/mouse IgG (Abclonal, anti-rabbit: AS014, anti-mouse: AS003) secondary antibody at room temperature for 1 h, and bands developed on the membrane using Syngene G-Box and GeneSnap software (Syngene, Cambridge, UK).

### Multicolor immunofluorescence

Formalin-fixed paraffin-embedded sections were fractionated alcohol dewaxed and rehydrated, and EDTA antigen retrieval buffer was subjected to antigen retrieval at 98°C for 8 min. Then the slides were soaked in 3% hydrogen peroxide for 15 minutes. CD63, CD168, α-SMA (Servicebio, Wuhan, Hubei, China) and FAT1 (Abcam, ab-190242) were applied at 4°C overnight, followed by incubation with secondary antibodies for 90 min at 37°C. Digital slide scanner (Pannoramic 250, 3DHistech, Hungary) were performed to scan samples.

### Animal xenograft tumor experiment

The animals used were 4-week-old female nude mice with BALB/c (Hfkbio, Beijing, China). In a specific environment, nude mice were randomly divided into two groups, control group and sh-FAT1 group (n=5). A total of 2×10^6^ cells were implanted subcutaneously in the right groin of nude mice, and when the tumor was clearly palpated, tumor volume measurement was started, and thereafter, tumor volume was measured every 2 days, and at the end of observation, the mouse subcutaneous tumor was excised, peeled and resected and images were collected. The tumor volume calculation formula used in this study is (L × W^2^)/2.

### Statistical analysis

R software was used in the statistical analysis. Student’s t-test was used to compare the expression levels of FAT1 between different groups, and the Wilcoxon rank-sum test was used to analyze non-normally distributed data. Pearson correlation analysis was employed to evaluate the correlation between FAT1 expression and immune infiltration.

## Results

### FAT1 is frequently mutated in NSCLC tissues

**Figure 1:**
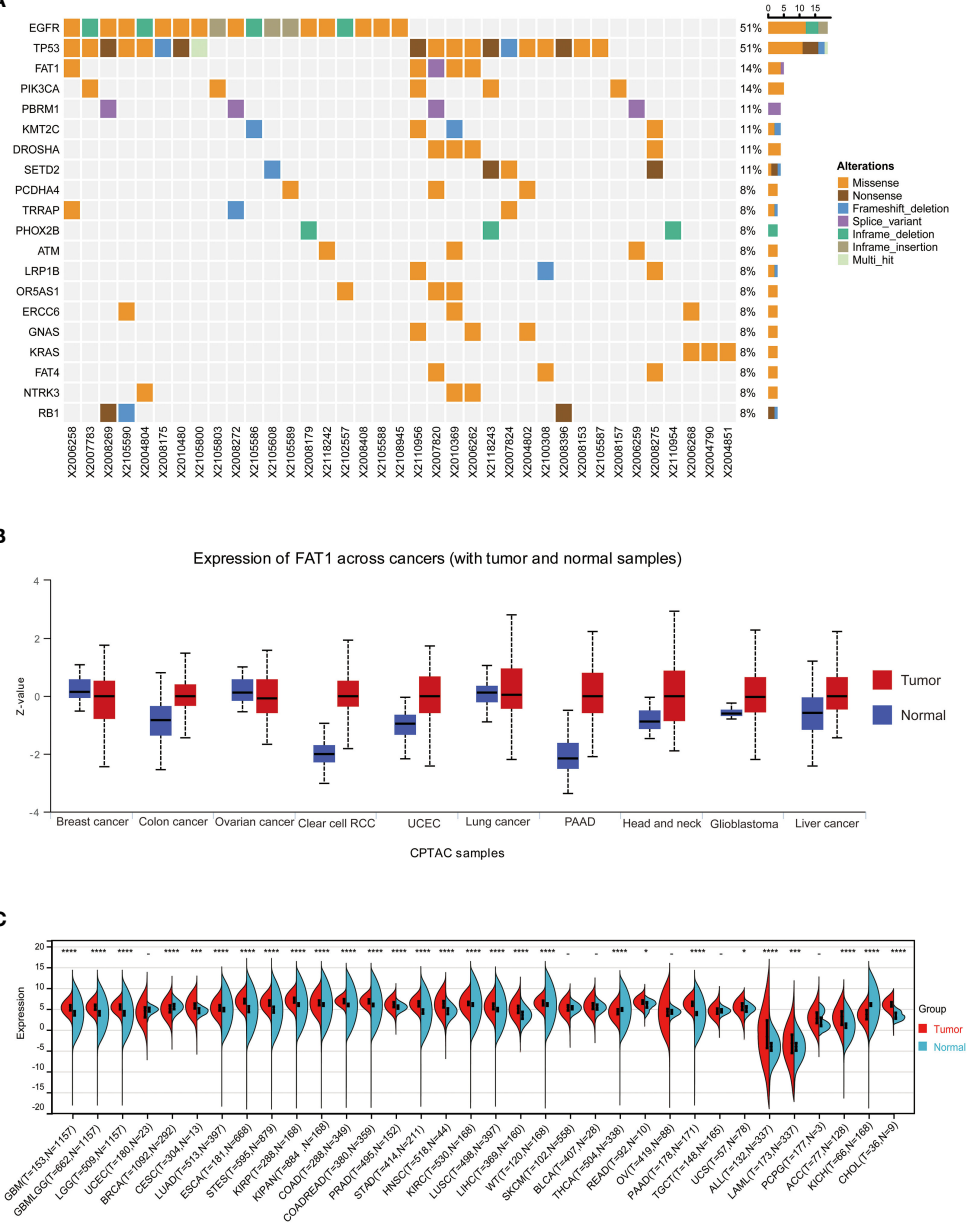
FAT1 expression in cancer. **(A)** Results of target gene capture second-generation sequencing in 37 lung cancer tissues. **(B)** FAT1 mRNA expression levels in various cancer types based on TCGA data. **(C)** Protein expression level of FAT1 in pan-cancer. *P < 0.05,***P < 0.001,****P < 0.0001.

Our research team previously established that FAT1 mutations may serve as predictive biomarkers for identifying NSCLC patients who may not derive sustained clinical benefits from ICB, thereby laying the groundwork for the potential application of individualized immunotherapy screening (10). In this present study, we first evaluated FAT1 mutation status in NSCLC patients. By collecting FFPE tumor tissues from 37 individuals who diagnosed with NSCLC and utilizing NGS technology for targeted gene capture, we analyzed a panel of 1267 genes for those patients. Our analysis identified the presence of FAT1 mutations in five out of thirty seven samples, constituting a mutation frequency of 14%. Among these mutations, four were missense mutations, while one was a splice variant (**Figure 1A**). Notably, within the 1267 genes analyzed, FAT1 ranked third in terms of mutation frequency, following EGFR and P53. This finding suggests that FAT1 may play a significant role in the pathogenesis and progression of NSCLC, warranting further in-depth investigation.

### FAT1 expression and its clinical significance in pan-cancer

**Figure 2:**
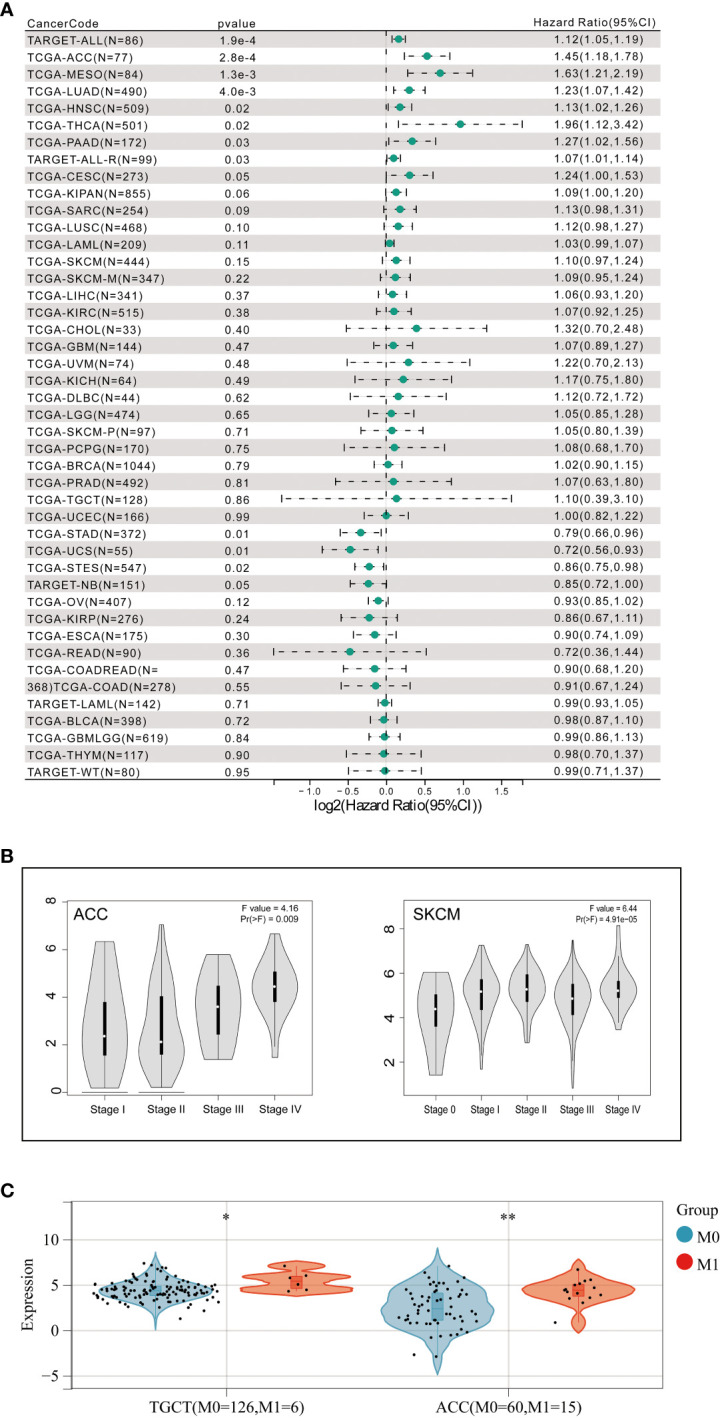
**(A)** Correlation between FAT1 expression and prognosis in various types of cancer using SangerBox. **(B)** Association of FAT1 gene expression levels with pathological stages and **(C)** metastasis. *P < 0.05,**P < 0.01

To further explore whether aberrant expression of FAT1 associated with clinical significance in pan-cancer, we conducted an extensive analysis of FAT1 expression across various cancer types, utilizing publicly available databases, including the TCGA and GTEx databases. Our findings align with prior reports (22), demonstrating a frequent upregulation of FAT1 expression in most cancer types when compared to their normal counterparts, including lung adenocarcinoma (LUAD) and lung squamous cell carcinoma (LUSC) (**Figure 1B**). Notably, protein expression analysis revealed significant variations in FAT1 expression across various tumors (**Figure 1C**), highlighting its potential role as a target in tumorigenesis and development. Furthermore, our investigation unveiled a substantial association between elevated FAT1 expression and adverse overall survival (OS) outcomes in patients with several tumor types, including acute lymphocytic leukemia (ALL), adenoid cystic carcinoma (ACC), mesothelioma (MESO), LUAD, head and neck squamous cell carcinoma (HNSC), thyroid carcinoma (THCA), pancreatic adenocarcinoma (PAAD), and cervical squamous cell carcinoma and endocervical adenocarcinoma (CESC) (**Figure 2A**; log-rank test, P < 0.05). Additionally, we observed a positive correlation between FAT1 expression and advanced pathological stages in ACC and Skin Cutaneous Melanoma (SKCM) (**Figure 2B**). Notably, metastatic testicular germ cell tumors (TGCT) and ACC exhibited higher FAT1 expression levels compared to their corresponding primary tumors (**Figure 2C**).

### DNA methylation analysis of FAT1 in pan-cancer

**Figure 3:**
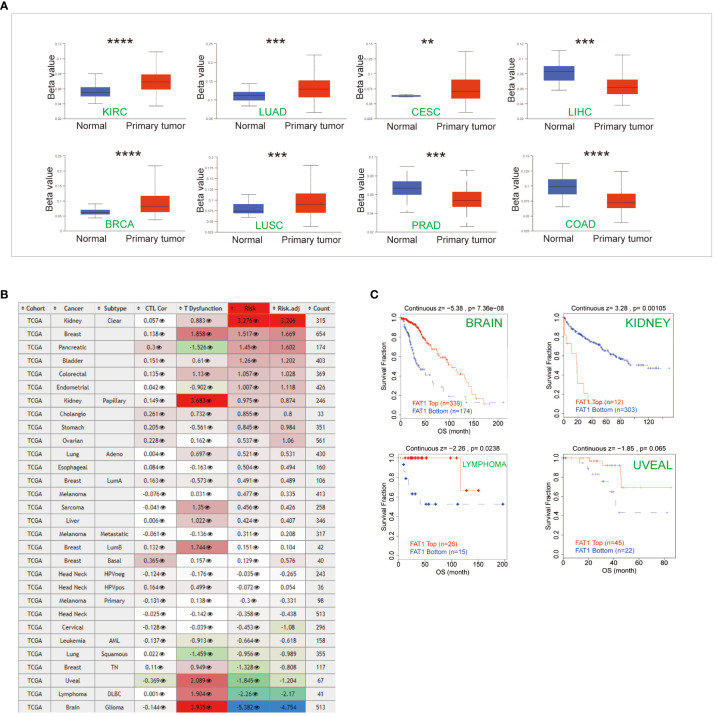
Epigenetic methylation analysis. **(A)** Boxplots illustrate the differential FAT1 methylation levels (beta values) across TCGA. **(B)** Heatmap demonstrating the effect of FAT1 methylation on cytotoxic T-cell levels (CTLs), dysfunctional T-cell phenotypes, and risk factors within TCGA cohorts. **(C)** FAT1 methylation levels at Kaplan-Meier survival curves. In kidney cancer, FAT1 hypomethylation is associated with a better prognosis, and in brain cancer, lymphoma, and uveal cancer, FAT1 hypomethylation has a shorter survival. **P < 0.01,***P < 0.001,****P < 0.0001

The promoter region of FAT1 displayed frequent hypermethylation in various cancer types, including kidney renal clear cell carcinoma (KIRC), LUAD, CESC, breast invasive carcinoma (BRCA), and LUSC. In contrast, the levels of FAT1 promoter methylation in Prostate adenocarcinoma (PRAD), liver hepatocellular carcinoma (LIHC), and colon adenocarcinoma (COAD) were lower when compared to their adjacent normal tissues (**Figure 3A**). This suggests that epigenetic silencing of FAT1 may play a role in its expression regulation in cancer. We conducted an in-depth analysis to examine the impact of FAT1 methylation on different cancer types and made intriguing observations. We found a correlation between FAT1 hypomethylation and dysfunctional T-cell phenotypes, as well as shorter survival in brain, lymphoma, and uveal cancers (**Figure 3B**). However, it’s noteworthy that in the context of kidney cancer, FAT1 hypomethylation was associated with a favorable prognosis (**Figure 3C**).

### Correlation analysis and pathway enrichment of FAT1 in pan-cancer

**Figure 4:**
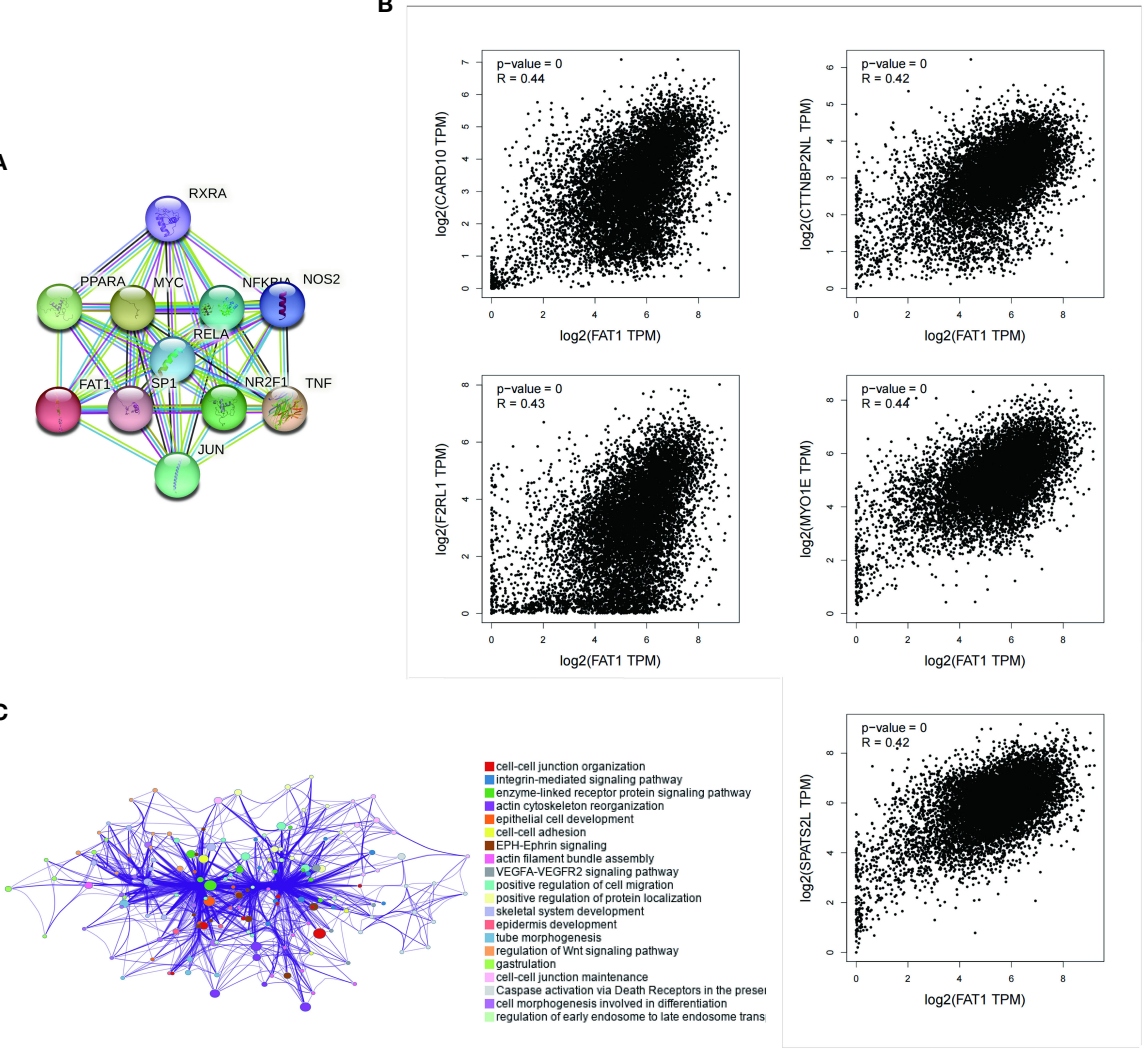
Enrichment analysis of FAT1-related genes across pan-cancers. **(A)** Displayed here are the FAT1-binding proteins identified using the STRING tool. **(B)** The top five FAT1-correlated genes are displayed across pan-cancers, and the relationships between FAT1 expression and these selected genes are analyzed using the GEPIA2 website. **(C)** Enriched terms with a similarity >0.3 are connected by edges.

We employed the STRING tool to systematically screen for FAT1-binding proteins, aiming to uncover the potential role of FAT1 in tumor pathogenesis. The interaction network reveals associations with RXRA, PPARA, MYC, RELA, NFKR, NOS2, SP1, NR2F1, TNF, and JUN (**Figure 4A**). To gain deeper insights into FAT1’s potential functions in cancer, we conducted correlation and pathway enrichment analyses using gene expression data from TCGA. Our analysis identified the top five genes significantly correlated with FAT1 expression: CARD10, CTTNBP2NL, F2RL1, MYO1E, and SPATS2L (**Figure 4B**). Additionally, an exploration of the top 100 FAT1-associated genes revealed significant associations with multiple cancer-related signaling pathways, encompassing cell–cell junction organization, integrin-mediated signaling pathways, and enzyme-linked receptor protein signaling pathways (**Figure 4C**).

### Distinct immune microenvironment based on FAT expression

Tumor-infiltrating immune cells wield a pivotal influence within the tumor microenvironment, impacting cancer initiation, progression, and metastasis (23, 24). In our prior study, we unveiled the potential of FAT1 aberrant as predictive biomarkers in NSCLC, indicating the potential role of FAT1 regulating immune microenvironment. To explore the connection between FAT1 expression and immune cell infiltration, we conducted a comprehensive analysis across 39 cancer types. Our investigation revealed a significant positive correlation between FAT1 expression and the infiltration of six immune cell types—namely, B cells, CD8+ T cells, CD4+ T cells, macrophages, neutrophils, and dendritic cells—in KIRC and Pheochromocytoma and Paraganglioma (PCPG) (**Supplementary Figure S1A**). Furthermore, we delved into the relationship between FAT1 expression levels and the infiltration of three immunosuppressive cell types known to promote T-cell exclusion: myeloid-derived suppressor cells (MDSCs), cancer-associated fibroblasts, and T-regulatory cells. Our findings indicated a positive correlation between FAT1 expression and the infiltration of MDSCs in several cancer types, including ACC, BRCA, BRCA-LumA, BRCA-LumB, Glioblastoma (GBM), Head and Neck Squamous Cell Carcinoma-Human Papillomavirus, Kidney Chromophobe, Kidney Renal Papillary Cell Carcinoma, and Low-Grade Gliomas (**Supplementary Figure S1B**). In our quest to assess FAT1’s relevance as a biomarker, we compared it to established biomarkers with regard to their ability to predict response outcomes and OS in ICB subcohorts. Notably, FAT1 exhibited an area under the receiver operating characteristic curve (AUC) exceeding 0.5 in 6 out of 18 ICB subcohorts (**Supplementary Figure S1C**). Additionally, FAT1 expression displayed positive correlations with immune-related gene signatures, encompassing immune cell infiltration, immune checkpoint genes, and major histocompatibility complex class I expression, suggestive of FAT1’s potential role in modulating the immune microenvironment of tumors. We conducted extensive correlation analyses between FAT1 expression and various genes, including chemokines and their receptors (e.g., CXC and CC family) as well as major histocompatibility complex classes I and II (**Supplementary Figure S2**). Among the 26 cancer types with high FAT1 expression, we noted elevated expression of CD274 (PD-L1), particularly in Diffuse Large B Cell Lymphoma (DLBCL), Uveal Melanoma (UVM), GBM, and PAAD. Furthermore, FAT1 expression positively correlated with PD-L1 and CTLA-4 expression in LUAD, suggesting the potential involvement of FAT1 in regulating the expression of immune checkpoint genes in lung cancer.

### Prediction of therapeutic response based on FAT1 expression

**Figure 5:**
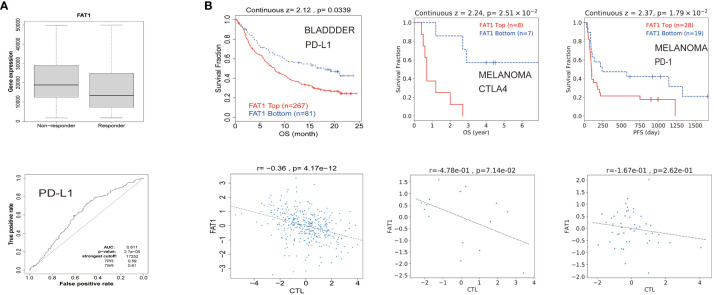
FAT1 expression is associated with therapeutic responses in cancers. **(A)** Receiver operating characteristic curve plot of the association between FAT1 expression and responses to PD-L1 in cancers. **(B)** The Kaplan-Meier survival curve showed shorter OS with high FAT1 expression in bladder cancer and melanoma receiving immunotherapy. The lower panel displays the correlation between FAT1 expression and cytotoxic T lymphocytes.

To explore the potential clinical utility of FAT1 as a biomarker, we conducted an assessment of its predictive value in gauging therapeutic responses among cancer patients. Our findings revealed a notable correlation between high FAT1 expression and an unfavorable survival to immune checkpoint inhibitors (**Figure 5A**). This observation suggests that FAT1 holds promise as a potential predictive biomarker for assessing responses to these treatments. Further analysis showed that elevated FAT1 expression was associated with shorter OS in patients treated with ICB for bladder and melanoma cancers. Moreover, heightened levels of FAT1 expression exhibited a negative association with cytotoxic T lymphocyte (CTL) (**Figure 5B**).

### Altered FAT1 expression changes tumor immune microenvironment

**Figure 6:**
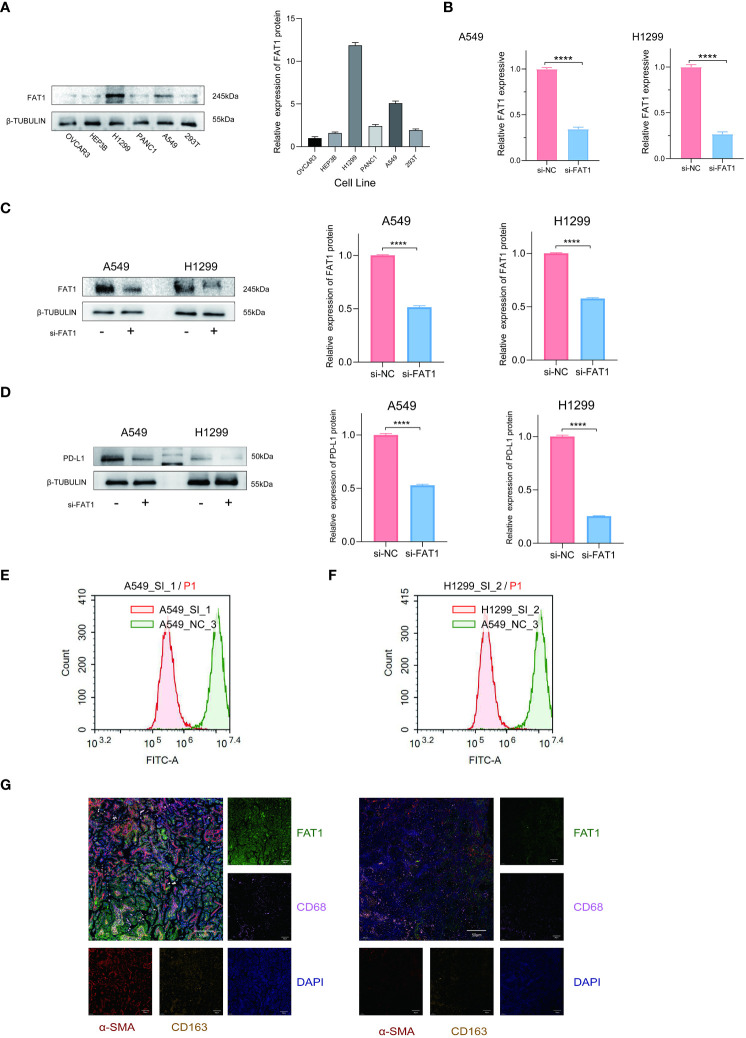
FAT1 knockdown effects in lung cancer cells. **(A)** Western blotting detects FAT1 expression in various cancer cell lines. **(B, C)** Western blot and qRT-PCR confirming FAT1 knockdown efficiency in lung cancer cells. Two-sided t-tests. **(D)** Western blot results verified the relationship between FAT1 expression and PD-L1. **(E, F)** Flow cytometry analysis to investigate the relationship between FAT1 and PD-L1 expression. **(G)** Multicolor immunofluorescence compared CD68, CD163 and α-SMA between FAT1 High (left) and Low (right) NSCLC tissue samples; ****P < 0.0001.

To explore the functional implications of FAT1 in the regulation of the tumor immune microenvironment, we initiated our investigation by assessing FAT1 expression levels across various cell lines, including OVCAR3, Hep3B, PANC1, H1299, A549, and 293T, utilizing Western blot analysis. Our findings revealed the highest expression of FAT1 in the A549 and H1299 cell lines, as illustrated in **Figure 6A**. Subsequently, we focused our *in vitro* experiments on these two lung cancer cell lines. To ensure the specificity and efficacy of the siRNAs targeting FAT1, we employed quantitative real-time polymerase chain reaction (qRT-PCR) and Western blot analyses. As expected, the siRNAs demonstrated significant reduction in FAT1 expression in both A549 and H1299 cells compared to the control group (**Figures 6B, C**). Cancer immunotherapy, with a particular emphasis on targeting the programmed death 1/programmed death ligand 1 (PD-1/PDL1) pathway, has exhibited remarkable therapeutic efficacy in lung cancer patients (25). The level of PD-L1 expression in tumor cells has emerged as a pivotal indicator of the effectiveness of PD-1/PD-L1 blockade (26), we elucidate the relationship between FAT1 expression and PD-L1. Our results indicated a substantial decrease in PD-L1 expression following the knockdown of FAT1 expression (**Figure 6D**). Flow cytometry analysis of PD-L1 expression also yielded similar results (**Figures 6E, F**). Recognizing the pivotal role of the immune microenvironment in tumor development (27), we further explored the correlation of FAT1 expression with myeloid-derived suppressor cells (MDSC), macrophage M1, macrophage M2, and cancer-associated fibroblasts (CAF) in lung cancer patient tissues through immunofluorescence assay. Intriguingly, our findings demonstrated a positive correlation between FAT1 expression and the markers CD68, CD163, and α-SMA, indicative of the essential role of FAT1 in immune regulation within NSCLC (**Figure 6G**). These results collectively underscore the critical involvement of FAT1 in orchestrating immune responses in the context of NSCLC.

### FAT1 accelerated the proliferation and migration of lung cancer cells *in vitro*

**Figure 7:**
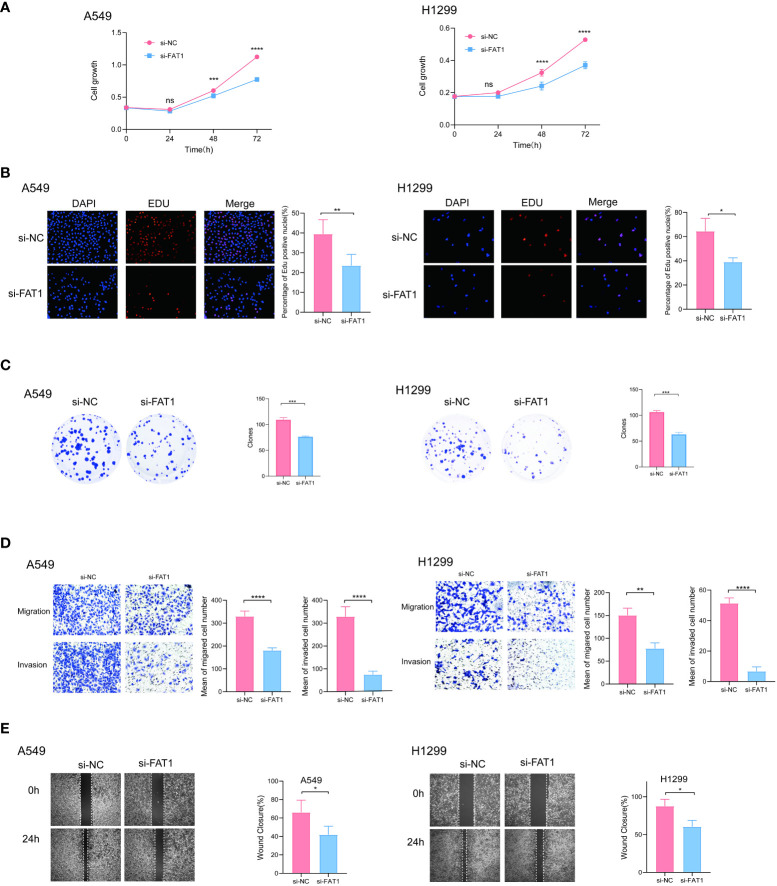
FAT1 knockdown effects in lung cancer cells. **(A)** CCK-8 assay measuring cell proliferation in FAT1-knockdown and control cells, two-sided t-tests. **(B)** EdU assay measuring cell proliferation in FAT1-knockdown and control cells, two-sided t-tests. **(C)** Colony formation assay showed that FAT1 knockdown inhibits the colony formation ability of cells. Two-sided t-tests; **(D)** Transwell migration and invasion assays showing decreased migration and invasion of FAT1-knockdown cells compared with control cells, two-sided t-tests. **(E)** Wound-healing experiments confirmed that knocking down FAT1 inhibits cell wound-healing ability, two-sided t-tests. ^ns^P ^>^0.05, *P < 0.05, **P < 0.01, ***P < 0.001, ****P < 0.0001.

**Figure 8:**
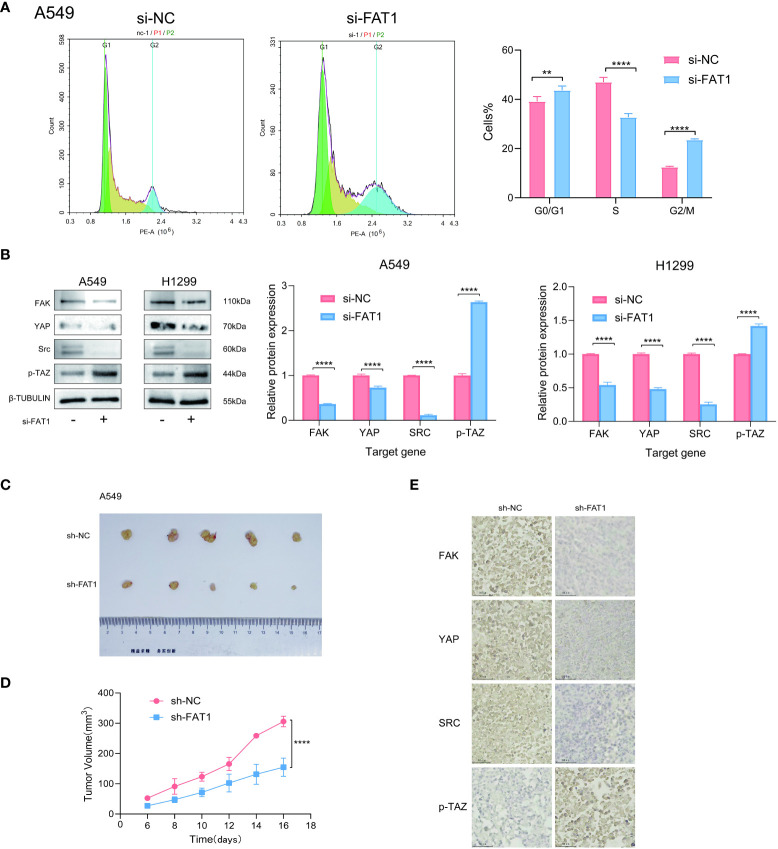
FAT1 knockdown effects in lung cancer cells. **(A)** Cell cycle assay showed that FAT1 knockdown induced G0/G1 phase arrest. **(B)** Western blot results verifying the effect of knockdown FAT1 on the FAK–YAP/TAZ signaling pathway. **(C)** Immunohistochemical validation of the effect of FAT1 knockdown on the FAK-YAP/TAZ signaling pathway. **(D, E)** Pictures of dissected mouse tumors and knocking down FAT1 inhibits tumor growth in mice. **P < 0.01, ****P < 0.0001.

To explore the biological role of FAT1 in NSCLC, we focused on investigating its effects on cell proliferation and migration. Employing the CCK-8 assay, we observed a pronounced inhibition of cell proliferation in both A549 and H1299 cells upon FAT1 knockdown, as evidenced by significantly reduced cell viability compared to the control group (**Figure 7A**). This anti-proliferative effect was further corroborated by the EdU assay, revealing a notable decrease in the proportion of EdU-positive cells following FAT1 knockdown, indicative of suppressed cell proliferation (**Figure 7B**). Furthermore, our investigation extended to colony formation assays, where FAT1 knockdown exhibited a significant impediment to both the number of colonies in both cell lines. These findings underscore the pivotal role of FAT1 in promoting not only lung cancer cell growth but also colony formation (**Figure 7C**). Delving into the realm of cell migration and invasion, the Transwell assay demonstrated a marked reduction in the number of migrating and invading cells in both A549 and H1299 cell lines following FAT1 knockdown, implicating FAT1 in the facilitation of lung cancer cell migration and invasion (**Figure 7D**). This observation was further supported by the cell scratch assay, where FAT1 knockdown attenuated the wound-healing ability of cells, indicating a weakened capacity for migration compared to the control group (**Figure 7E**). Moreover, our exploration revealed that FAT1 knockdown induced G0/G1 phase cell cycle arrest (**Figure 8A**). To shed light on the underlying molecular mechanisms, Western blot experiments targeting integrin-related pathways were conducted based on pathway enrichment results. The outcomes suggested that knocking down FAT1 may exert its effects on cell growth and proliferation through the FAK–YAP/TAZ pathway (**Figure 8B**). To further validate the impact of FAT1 on the FAK-YAP/TAZ pathway, immunohistochemical staining was performed on mouse tumor tissues, revealing consistent results (**Figure 8E**). This intricate interplay emphasizes the multifaceted involvement of FAT1 in orchestrating cellular processes crucial for the progression of NSCLC.

### Knockdown of FAT1 inhibited the proliferation of lung cancer cells *in vivo*

To further confirm the effect of inhibiting FAT1 expression on cell proliferation observed *in vitro*, we conducted xenograft tumor experiments to assess the effect of FAT1 knockdown on lung cancer cell growth *in vivo*. The results demonstrated that knocking down FAT1 inhibited the growth of lung cancer cells *in vivo* (**Figures 8C, D**). These comprehensive findings collectively underscore the role of FAT1 as a tumor promoter in lung cancer cells, with its downregulation leading to the inhibition of cell proliferation.

## Discussion

FAT1, a sizable transmembrane protein, plays a significant role in diverse biological processes, encompassing cell adhesion, migration, and proliferation (28, 29). Extensive research has unveiled frequent upregulation of FAT1 in numerous cancer types, including lung cancer, suggesting its potential as a pivotal contributor to tumorigenesis (30, 31). Consequently, FAT1 emerges as a promising candidate for both therapeutic intervention and prognostic assessment in cancer treatment. Under normal circumstances, FAT1 functions as a molecular ‘brake’ on mitochondrial respiration and serves as a receptor involved in the regulation of cell–cell contact interactions and planar cell polarity (22, 29, 32). In several cancer types, the loss of FAT1 function contributes to epithelial-mesenchymal transition (EMT) and the emergence of cancer-initiating or stem-like cells (8, 33–35). Conversely, in specific cancer types, FAT1 overexpression induces EMT (30). However, the precise roles of FAT1 in cancer progression remain intricate and contingent on the specific cancer type. Consequently, further investigations are warranted to attain a comprehensive understanding of FAT1’s function within distinct cancer contexts. In this study, we conducted a comprehensive investigation into the expression patterns and clinical implications of FAT1 across a spectrum of cancer types employing bioinformatics analysis. Additionally, we substantiated its pro-tumorigenic role through experimental validation in lung cancer cells. Our findings, in alignment with prior research, consistently demonstrated a prevalent upregulation of FAT1 expression in a majority of cancer types, including LUAD and LUSC. Notably, we established a significant association between elevated FAT1 expression and reduced OS in LUAD patients, thus hinting at the potential prognostic utility of FAT1 in lung cancer. Furthermore, our study revealed a positive correlation between FAT1 expression and advanced pathological stage as well as metastasis in various cancer types, providing additional substantiation for the putative role of FAT1 in tumor progression and metastasis. Additionally, we illuminated potential mechanisms underpinning FAT1 upregulation in cancer, such as promoter hypermethylation—a phenomenon observed across multiple cancer types. Interestingly, our investigation also unveiled a positive correlation between FAT1 expression and immune-related gene signatures, suggesting a potential involvement of FAT1 in modulating the immune microenvironment within tumors. Furthermore, our analysis revealed a distinctive set of genes and signaling pathways that exhibited significant correlations with FAT1 expression, thereby providing additional insights into the potential functional roles of FAT1. Remarkably, our investigation unveiled FAT1’s association with pathways linked to tumor occurrence and development, including cell–cell junction organization, the integrin-mediated signaling pathway, and the enzyme-linked receptor protein signaling pathway. Integrin-mediated cell migration predominantly relies on the activation of the FAK/Src signaling pathway, which, in turn, contributes to the regulation of several key signaling cascades governing cell motility (36). Noteworthy, previous studies have identified YAP/TAZ as downstream molecules of the Hippo signaling pathway, exerting control over cell proliferation and apoptosis, thus playing a pivotal role in tumor growth regulation. Integrin-FAK/Src activation has been shown to enhance YAP activation, leading to the accumulation and activation of YAP/TAZ, further promoting the proliferation and metastasis of malignant tumors (37, 38). Additionally, our assessment of the correlation between FAT1 expression and immune checkpoint genes hints at the potential utility of FAT1 as a predictive marker for immunotherapy efficacy. To gain deeper insights into FAT1’s implications in cancer, we conducted *in vitro* functional assays utilizing lung cancer cell lines. Targeting FAT1 expression through knockdown in these cells resulted in a notable reduction in cell proliferation, migration, and invasion, accompanied by cell cycle inhibition. These findings strongly substantiate an oncogenic role for FAT1 in lung cancer and suggest that targeting FAT1 may hold promise in enhancing the effectiveness of chemotherapy in lung cancer treatment.

In conclusion, our study conducted a comprehensive bioinformatics analysis to investigate the functional role of FAT1 across diverse cancer types, with a particular focus on lung cancer. The insights derived from our findings underscore the potential utility of FAT1 as both a biomarker and a therapeutic target not only in lung cancer but also in various other cancer types.

## Data availability statement

The original contributions presented in the study are included in the article/**Supplementary Materials**. Further inquiries can be directed to the corresponding authors.

## Ethics statement

The studies involving humans were approved by Institutional Ethics Committee of the General Hospital of Tianjin Medical University. The studies were conducted in accordance with the local legislation and institutional requirements. Written informed consent for participation in this study was provided by the participants’ legal guardians/next of kin. The animal study was approved by Ethics Committee of Tianjin Medical University General Hospital. The study was conducted in accordance with the local legislation and institutional requirements.

## Author contributions

CD: Validation, Writing – review & editing. HH: Writing – review & editing. DW: Data curation, Writing – original draft. CC: Writing – review & editing. YH: Writing – original draft. JL: Funding acquisition, Writing – review & editing. YL: Methodology, Writing – review & editing. HL: Methodology, Writing – review & editing. JC: Methodology, Writing – review & editing.
