# Supplementary material for: Pan-cancer analysis predict that FAT1 is a therapeutic target and immunotherapy biomarker for multiple cancer types including non-small cell lung cancer
Source: Front Immunol. 2024 May 24;15:1369073. doi: 10.3389/fimmu.2024.1369073 (PMC11157030; doi:10.3389/fimmu.2024.1369073)
Supplement: Supplementary file 1 [file DataSheet_1.docx]

**
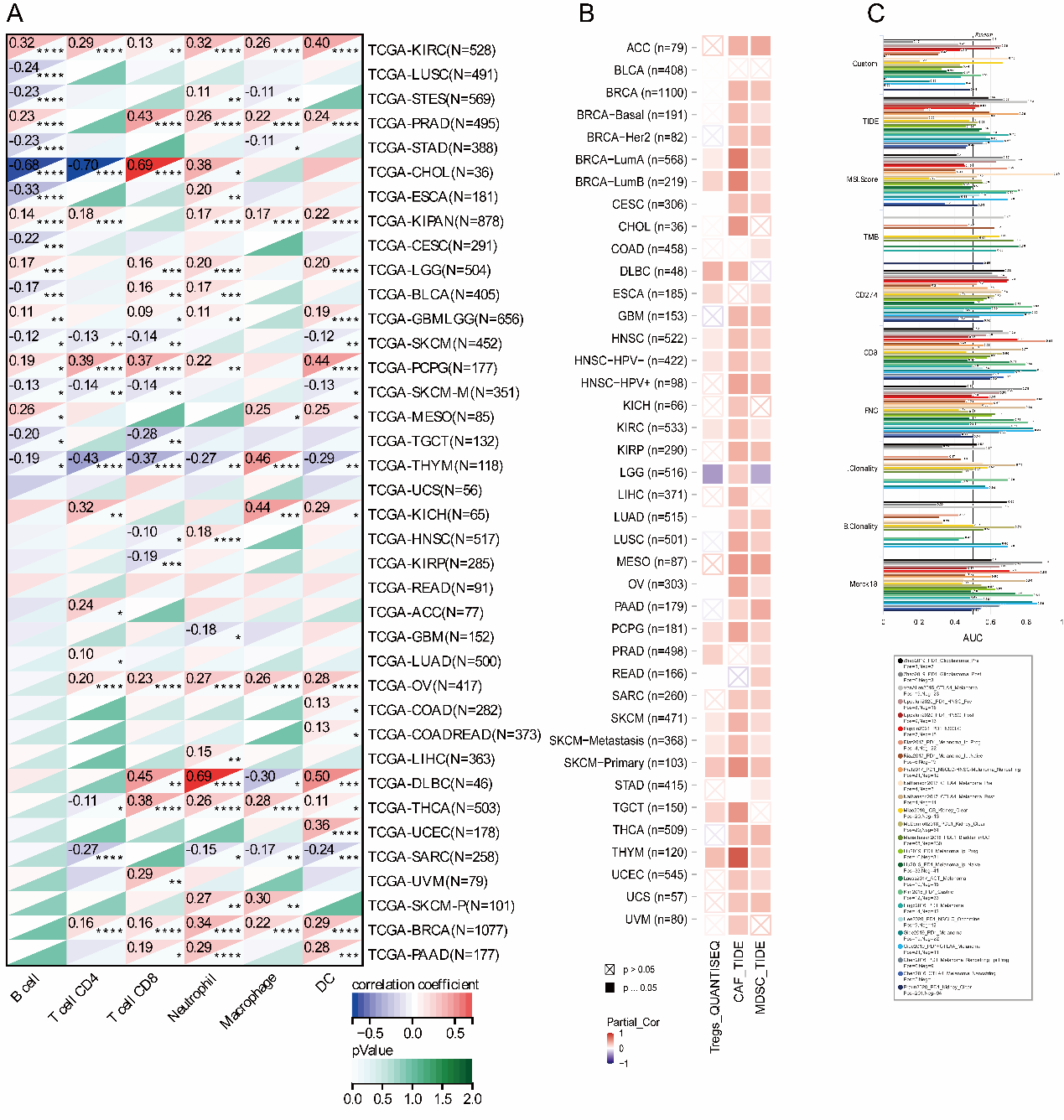
**

**Figure S1**: Heatmap of the correlations between FAT1 expression and infiltration levels of (A) six immune cell types and (B) three immunosuppressive cell types in TCGA cohorts. (C) A bar plot comparing the biomarker relevance of FAT1 with standardized cancer immune evasion biomarkers in immune checkpoint blockade (ICB) subcohorts. cancer-associated fibroblasts (CAFs), myeloid-derived suppressor cells (MDSCs), and regulatory T cells (Tregs).


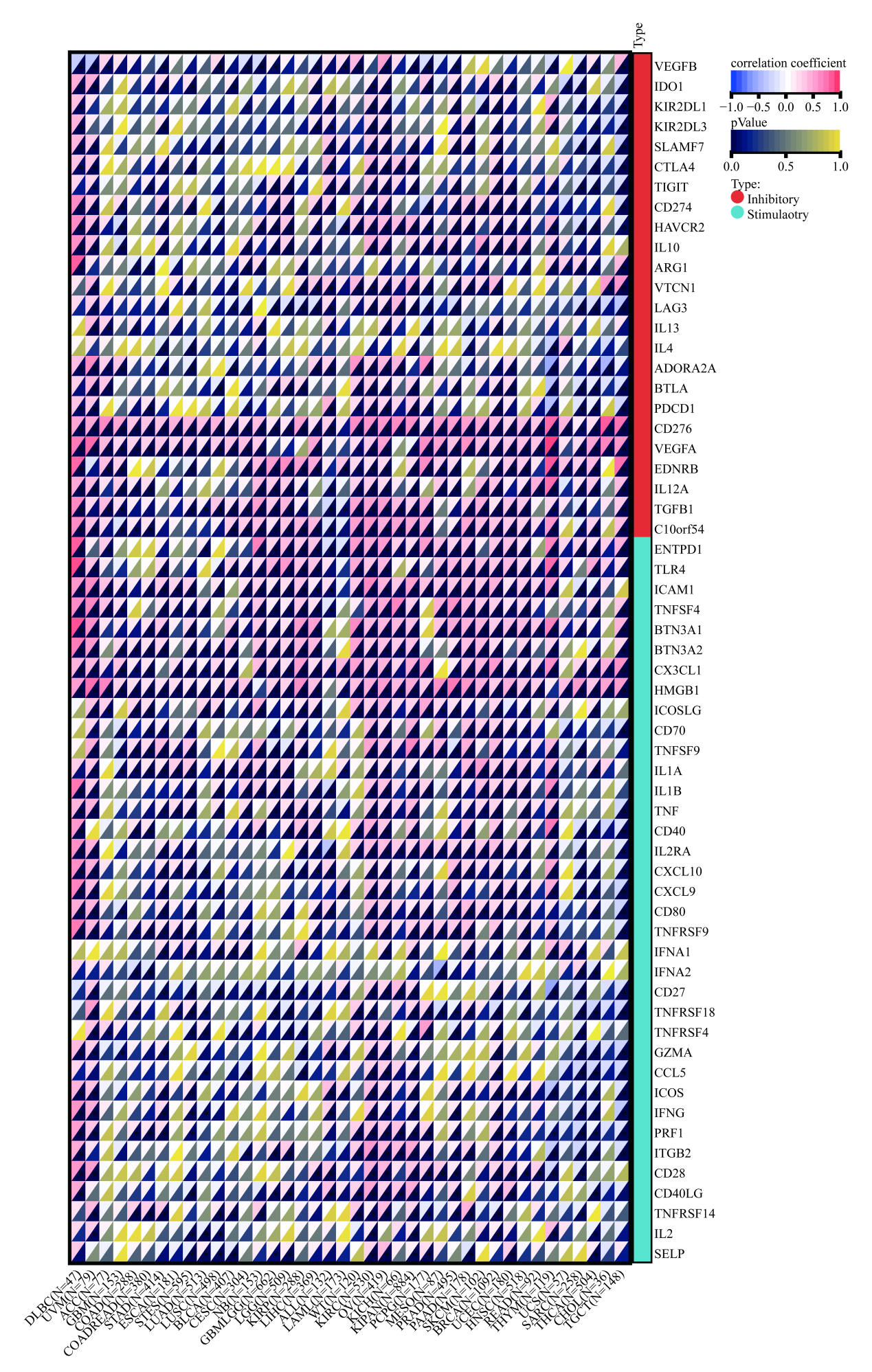


**Figure S2**: Relationship between FAT1 mRNA expression and immune check points in multiple cancers by SangerBox online website.
